# ‘Fleeing’ as a Strategy for Navigating Resistance in Patient Encounters within Forensic Care

**DOI:** 10.3390/healthcare11212890

**Published:** 2023-11-02

**Authors:** Lars Hammarström, Ove Hellzén, Siri Andreassen Devik

**Affiliations:** 1 Department of Nursing Science, Mid-Sweden University, 851 70 Sundsvall, Sweden; lars.hammarstrom@miun.se (L.H.); ove.hellzen@miun.se (O.H.); 2 Centre for Care Research, Mid-Norway, Faculty of Nursing and Health Sciences, Nord University, 7800 Namsos, Norway

**Keywords:** nurse–patient interactions, emotional regulation, forensic psychiatry, reflective lifeworld research

## Abstract

The aim of this study was to describe the phenomenon of “fleeing the encounter when facing resistance” as experienced by carers working in forensic inpatient care. Qualitative analysis, namely reflective lifeworld research, was used to analyze data from open-ended questions with nine carers from a Swedish regional forensic clinic. The data revealed three meaning constituents that describe the phenomenon: shielding oneself from coming to harm or harming the other, finding one’s emotional balance or being exposed, and offering the patient emotional space and finding patience. The carers described their approaches in the encounters with the patients as alternating between primitive instincts and expectant empathy in order to gain control and deal with the interaction for their own part, for that of the patient, and for that of their colleagues. The phenomenon of fleeing the encounter when facing resistance was intertwined with carers’ self-perception as professional carers. Negative encounters with patients evoked feelings of shame and self-blame. A carer is a key person tasked with shaping the care relationship, which requires an attitude on the part of the carer that recognizes not only the patient’s lifeworld but also their own.

## 1. Introduction

Forensic psychiatric care differs from other care in that it is a legal sanction for a person who has been convicted of a criminal act but who, instead of prison care, is handed over to forensic psychiatric care due to a serious mental disorder. This means that security and restrictions on freedom, etc., are governed by regulations based on the risk of recidivism [1]. At the same time, care must be governed by the person’s need for interventions for the condition from which they suffer. Interaction between patients and staff is important in the care of patients in forensic psychiatry [2]. Healthcare has an extra responsibility to ensure that the treatment, nursing care and management, and rehabilitation provided within the framework of coercive legislation are of a consistently high quality. The Swedish National Board of Health and Welfare [3] has issued instructions regarding safety in psychiatric compulsory care. Security is divided into three levels—very high (i.e., buildings must withstand qualified escape attempts and exemption attempts), high (i.e., escape-resistant), and acceptable (i.e., constant patient monitoring). The research on the relationship between housing climate and aggressive behavior emphasizes the importance of staff achieving a balance between structure and adaptability, while maintaining a supportive, responsive, and non-punitive approach to patients [4]. The content of the care must also be equivalent to the care offered in other parts of psychiatry and all treatments must be based on current knowledge [5]. In forensic psychiatry, the challenge is to meet patients’ emotional needs, set boundaries and connect, and be responsive and caring [6].

According to the Swedish Municipalities and County Councils [5], all 20 Swedish forensic psychiatry units work actively with prevention around the area of threats and violence. The majority of the work is based on the Bergen model, which originated from the Norwegian TERMA training model, developed at the Haukeland University Hospital, Department of Forensic Psychiatry in Bergen, Norway [7]. The method is a model for behavioral support, developed by McDonnell [8], that aims to prevent and respond to threats and violence in order to create an environment that works for both patients and staff. The model’s focus is to identify affect as an obstacle and focus on how people with problematic behaviors are treated in crisis situations, thereby creating good relationships between patients and staff.

Both patients and carers are particularly exposed to incidents of violence in forensic psychiatric institutions [9]. The consequences include physical and psychological damage to fellow patients and carers, poor therapeutic relationships, low job satisfaction, and increased sickness absence among staff [10,11]. Dealing with patients’ physical and verbal outbursts is considered a part of nurses’ everyday work in forensic care [12] and makes them vulnerable to burnout [13]. Being exposed to stressful situations, characterized by threats, violence, and harassment by patients, over long periods means that nurses often have to deal with emotions of frustration, fear, humiliation, disappointment, etc. [14]. According to Hochschild [15], managing one’s emotions is crucial when working with a focus on other people, a process that is described as emotional labor. Gross [16] believes the strategy for acting early in this process is emotional regulation. According to Løgstrup [17], all humans have an untouchable zone that is closely related to emotions that are difficult to manage and have an ethical meaning. The untouchable zone relates to the integrity of a person and is, therefore, violated if touched. The strategy of emotional regulation involves suppressing or re-evaluating one’s feelings [18]. The above-mentioned Bergen model is strongly influenced by the city model [19]. The city model is a theoretical nursing-based framework that is considered vital to reducing conflicts and containment in psychiatric wards, which emphasizes the self-regulation of emotional responses, including an awareness and control of one’s feelings, especially of fear and anger [19].

Controlling one’s own emotions can be understood as a strategy for managing and minimizing problematic behavior [8]. According to Hammarström et al. [20], nurses regulate their emotions in a forensic psychiatric environment as a way of dealing with emotions, not only in relation to themselves and the patient’s expression of suffering, but also in relation to other nurses and patients (i.e., to preserve the institution’s stability as a way of creating conditions for a caring climate). Regulating oneself as a way of striving for control means recognizing one’s shortcomings and vulnerability as a prerequisite of getting to know the patient, getting closer to them, and establishing an alliance and a trusting relationship. Regulating oneself entails more than just introspection; it also involves recognizing and addressing conflicting emotions without displaying the ongoing internal conflict. In other words, this means challenging one’s understanding of oneself and one’s preconceived notions to be open to the patient’s world of life [21].

Research has shown that the need for regulating emotions is central when working in the service sector, for example, in the health sector [15,16]. In psychiatric care, a need for regulating one’s own emotions usually occurs in connection with staff experiencing emotional reactions, such as fear and anger, in connection with conflicts with patients [19]. Hammarström et al. [21] have described nurses’ handling of emotions in such situations as coping strategies. Among the strategies identified was ‘preserving oneself as a carer’, which included ‘fleeing from situations’ as a way of coping with emotions when faced with resistance or feeling threatened. This perspective seems to be a crucial focal point for generating knowledge that could offer deeper insights into the Bergen model or other low-affective approaches. Such insights could play a pivotal role in developing more targeted care methods aimed at enhancing forensic psychiatric care and promoting patient participation in their care and recovery.

Moreover, within the context of forensic psychiatric inpatient care, the everyday social interactions between carers and patients can give rise to negative psychosocial outcomes [22]. During such instances, how nurses respond to patient expressions becomes of paramount importance. Recently, a systematic review showed limited evidence that training staff in de-escalation techniques is effective in reducing verbal and physical aggression in forensic patients [23]. However, interventions that promote the establishment of therapeutic relationships and individualized patient-directed care appear to yield more promising results [24]. Nevertheless, the strategies used by carers when confronted with challenging patient behavior in forensic treatment remain largely unexplored [20]. The same applies to knowledge about how carers navigate their own emotions in general during interactions with patients in this context [25].

Björkdahl et al. [7] succinctly categorize carers’ coping strategies into confrontational or avoidant approaches, while Hammarström et al. [21] reveal that carers, when overwhelmed by their own emotions, sometimes opt to distance themselves from patient encounters, a tactic that not only separates them from the patient but also creates a divide from their own fears or frustrations. This inability to consistently manage their emotions during patient interactions can be assumed to have implications for staff well-being, staffing stability, as well as the quality of patient care [26]. Therefore, this study aims to elucidate the phenomenon of “fleeing the encounter when facing resistance” as experienced by carers in forensic inpatient care.

## 2. Materials and Methods

### 2.1. Design

To gain access to the carers’ lived experience of fleeing the encounter when facing resistance [18], a reflective lifeworld strategy [27] was chosen based on Edmund Husserl and Maurice Merleau-Ponty’s phenomenological epistemology. Reflective lifeworld research aims to clarify and illuminate the structure of meanings of phenomena related to human existence [27,28]. Through this approach, the goal was to come as close to the essential meaning and its variations as possible and thereby further develop the understanding of the phenomenon of fleeing the situation in patient encounters.

The leading principle for the chosen approach is an understanding of the world and the body as lived and experienced as something focused on meaning as well as reversibility. Such research demands a phenomenological attitude, which is characterized by openness to the lifeworld phenomenon, ongoing reflection upon the meanings, the bridling of understanding, i.e., a metaphor for describing what phenomenological researchers should implement with regards to their assumptions and preconceptions, because they aim to either describe or interpret phenomena, and move between distance and closeness [27,28,29]. In concrete terms, this meant that we had to focus on how the informants perceived, interpreted, and reacted to what was happening in the patient encounter and in their relationship with the patient. To succeed in this, a phenomenological attitude was required. In other words, we had to discard any assumptions we may have held about the sought phenomenon based on our previous research, knowledge, and experiences in forensic psychiatry inpatient care. Our intention was to gather as rich descriptions as possible of the informants’ experiences of fleeing the situation in connection with the patient encounter. This necessitated our concentration on how the phenomenon, in all its complexity, appeared in the interviews with the carers.

### 2.2. Procedure and Setting

The clinic manager approved this study. Department managers passed on information and invitations to employees who met the inclusion criteria (having experience of forensic care). Participants who were willing to participate contacted the researchers and received further information about the conduct of the study. All participants gave written consent before the interviews were conducted. The first author who performed the interviews was known to some of the participants, either as a former colleague, researcher or supervisor.

The clinic is one of Sweden’s regional clinics and one of the larger clinics with national admissions. The facility was classified as a high-security clinic, with one department classified as very high security. The clinic was populated by approximately 180 employees and 100 inpatients (spread over eight wards) at the time this study was conducted in winter 2021. The patients at the wards were mostly men between the ages of 25 and 45, with the majority suffering from severe mental disorders and transmitted to involuntary inpatient forensic psychiatric care due to a crime in accordance with the statute “The Forensic Mental Care Act” [30], rather than incarceration within the penal system. All ward staff at the clinic since 2017 had undergone a training program for violence prevention, RESIMA (resources in the face of aggression), inspired by the Bergen model [7] with a focus on low-affective prevention and managing the climate in the clinic’s care departments. The RESIMA model is a method for preventing and responding to threats and violence in psychiatric wards. The goal is to create a care environment that works in the best possible way for both patients and staff that is intended to permeate both the values and the care available at the studied forensic clinic. The focus is on preventive work in everyday life with good relations between patients and staff. The model defines clear guidelines for how staff should act in connection with threats and violent incidents and the starting point is that both patients and staff should feel safe and secure in psychiatric care [7].

In the training program, the metaphors “bulldozer” or “ballerina” are frequently used to describe the approaches used in encounters between carers and patients, metaphors described in Björkdahl et al. [7]. For example, the ‘Bulldozer approach’ is characterized by a carer who takes on the role of a protective power shield in the encounter with the patient with the intention of protecting what he or she considers to be a safe and secure ward for patients and staff. The ‘ballerina approach’ refers to cultivating a sense of security, trust, and closeness between oneself and the patient by talking to the patient, setting aside time, and being open and available. In the interviews, carers used these metaphors frequently to describe their approaches used in the patient encounter (see subsection findings).

### 2.3. Participants and Data Collection

In our efforts to achieve a diverse participant selection, we enlisted the assistance of the clinic’s management to distribute invitations based on a staff demographic that would ensure a variety of gender, age, and professional experiences within the field of forensic inpatient psychiatry. The only explicit inclusion criterion was that participants must have a minimum of 2 years of experience in forensic inpatient care. Nine individuals (comprising four women and five men) provided written consent to participate. The participants ranged in age from 30 to 66 years (Md = 41.6 years) and had experience in forensic inpatient care ranging from 2 to 32 years (Md = 12 years). Among these participants, five were specialist nurses in psychiatric care, one was a registered nurse, and three were assistant nurses. In this study, to conceal the participants’ identities, participants are referred to as “carers” based on the fact that caring could be seen as part of our common human moral compass regardless of education [31].

The first author conducted all interviews digitally with video and audio recording, as restrictions due to the COVID-19 pandemic prohibited the author from personally visiting the participants. The interviews lasted between 39 and 51 min (Md = 44 min). The interviewees were initially asked to describe a problematic everyday encounter, the patient’s actions, and their own feelings. In the rest of the interview, the main questions were as follows: Can you talk about one or more encounters that have been difficult to handle? What happened? How did you feel in the situation? How did you act? What were your feelings afterwards? Follow-up questions were asked to encourage the participant to elaborate and share more with the intention of obtaining as rich descriptions of the sought phenomenon as possible [32]. Participants were asked to share their narratives about their lived experience of fleeing the situation to regulate themselves when encountering patients in forensic inpatient care.

### 2.4. Data Analysis

The interviews were transcribed verbatim by the first author and the text was analyzed for meaning in relation to the aim of the study. Once acquainted with the text, the authors endeavored to enhance their comprehension of the data by partitioning the text into meaning units following the structure of whole-parts-whole, as it is used and described by Dahlberg et al. [27], and clusters were formed. These clusters were discussed and reflected upon by all authors, thereby elucidating the phenomenon’s essential meanings and overall structure. An example of the analysis process is shown in Table 1. During this part of the analysis, the importance of bridling one’s pre-understanding and keeping an open mind, avoiding making definite what is indefinite, became even more apparent. Bridling one’s pre-understanding means restraining one’s pre-conceived notions and problematizing one’s natural attitude, allowing the phenomenon’s multiplicity to show itself, as “it is more than meets the eye” [27]. Discussions among the authors made bridling possible. During these discussions, bridling one’s naturalistic intentions was a continuous challenge, especially for the first author, in this sense that the co-authors’ different backgrounds and prior knowledge was an asset in the numerous discussions to facilitate the naturalistic intentions allowing the phenomenon to show itself [33]. The first author’s familiarity was an asset, as was the co-authors’ objectivity. The co-authors were more well-acquainted with home care for the elderly, but, in both cases, they did not have updated clinical experience of the studied context. A new whole structure of the essential meanings of the phenomenon of fleeing the situation as an aspect of emotional regulation was formulated and further described by its constituents, which are the experiential variations of the phenomenon [27].

## 3. Results

In this study, “fleeing the encounter when facing resistance’ meant a strive for control in relation to oneself as a carer as well as to the patient. The meaning constituents represent the variations of the meaning: shielding oneself from coming to harm or harming the other, finding one’s emotional balance or being exposed, and offering the patient emotional space and finding patience.

Fleeing the encounter is shown as a concept with a relational and embodied nature. Carers described stepping back from the situation as a relational act, as it presupposes one’s openness to exposure to the patient, and it depicts the inherent uncertainty that exists in the relationship. The essence of the searched aspect of the phenomenon of fleeing the encounter means struggling between acting instinctively primitive and expectantly empathic. The essence of fleeing the encounter is described by the carers as trying to understand oneself and the situation that has arisen. Looking inwards, with the ambition of reflecting on the situation and one’s behavior, is perceived as demanding. Encountering the patient’s expressions evokes emotions that force the carer to act by fleeing the encounter. When being provoked emotionally and acting on this basis, the carer tries to handle the situation based on interpretation and understanding. When interpreting a patient’s expressions, having prior work experience means being less naive in the encounter with a provoking patient. Being in the middle of the action and making a quick decision on how to act means standing alone with the patient and being both physically and emotionally isolated in the situation. Contact with oneself is important for one’s understanding of what it means to be fragile in a context that is perceived as threatening and harmful.

### 3.1. Shielding Oneself from Coming to Harm or Harming the Other

Working in forensic psychiatric care means being confronted with patients who have committed serious crimes, such as murder, manslaughter, aggravated assault, rape, and/or sexual abuse of children, something that makes it difficult to approach the patient. Fleeing the encounter is characterized by the tension between being afraid and having courage. This aspect of the phenomenon of fleeing the encounter means struggling between finding one’s boundaries and having the courage to face the patient. In their stories, carers describe that they become aware of how powerful and infectious the patient’s actions are—they affect the entire staff group and are something that one cannot be defended against. When a threatening situation arises, the fight or flight instinct takes over. However, this primitive reaction must be regulated to ensure it does not take over the situation.

*I can feel it myself when someone comes with aggression, no matter who it is. These are very strong emotions, and it affects you so much. It is important to slow down the adrenaline so that the caveman does not take over*.

The critical point in the encounter is described when the carer does not know where the line between care or custody falls and fear takes over. Carers express that they sometimes do not successfully manage their emotions; instead, they have to leave the situation and the patient. In this case, fleeing the situation and being isolated from the patient means an opportunity to critically reflect, which reduces feelings of failure and fleeing from being a bulldozer. Carers said, “one is just a human being and cannot stand everything.” In such situations, the carer stands alone, face to face with the patient. The carers stated that this sore spot is a constant companion in the relationship with all patients and it is described as something that everyone will become aware of eventually.

*For my part, I would still like to say that the single biggest factor for me that is most difficult to deal with when it comes to affect regulation is fear. If I experience the situation, if I actually get scared, it is for me the absolute most difficult affect to deal with. It’s an affect that you almost can feel physically… throughout the whole body. You just want to escape and survive*.

Carers express the need for a strategy that allows them to take a step back, a way of thinking that help them to continue to feel professional. This strategy helps them to have enough energy to be able to control their own emotions when needed. Even if the carer knows what the patient needs and how they should act in the situation, it is sometimes difficult to meet the requirements when fear arises. Instead, they choose to release contact with the patient and take a step back from the encounter. The decision to abandon the arising situation or not is based on work experience. They judge that the patient poses a threat to themselves, and that the situation can suddenly become uncontrollable, which arouses fear in the carers. The fear affects the carers’ actions, which leads to conflicting experiences that make them deal with the situation that has arisen by fleeing it.

Sometimes, when the fight or flight instinct takes over, the carer acts by attacking the patient, by acting as a bulldozer. On such occasions, the patient often reacts with violence, which may result in the patient having to be secluded. Often, the carer’s reaction depends on his/her well-being. One carer said, “It’s about how I feel myself. If I feel bad, then I can’t cope with the patient”.

### 3.2. Finding One’s Emotional Balance or Being Exposed

This meaning constituent refers to the struggle between being distanced, like a bulldozer, or compassionate and caring, like a ballerina. Fleeing the encounter is characterized by a tension between the conflicting emotions of feeling knowledgeable and competent vs. feeling ignorant and incompetent, and this tension is connected to feelings of insecurity in one’s professional role. A tension that affects carers’ abilities to manage patient encounters lies between being shocked or being unaffected. The experience of and need for emotional balance is existential and causes the carer to take a step back from the situation and reflect on the arising situation to avoid ending up in an unmanageable situation. If not, they risk being “revealed” and their insecurity and feelings of incompetence will become known. At this point, it is easier to be commanding and relate to rules, to listen to the bulldozer, than to show their vulnerability in the situation.

Knowing the patient and having an established relationship is important. The relationship provides knowledge about the patient and makes it easier to predict the patient’s actions, which helps the carer deal with their fears or frustration. The more information the carers have about the patient and their previous actions, the easier it is for carers to predict how the situation will develop. The carers emphasize the importance of not showing the patient fear to manage the situation and prevent the fear from spreading.

*To let that adrenaline come and just accept the fear, and just try to continue to be who you are, and not show how scared I was. I have to take a step back for a while. If other patients see me scared, there is a risk that they will be worried. I guess that was what I did, but it was difficult. Because he was so sick and scary.*

When situations of threats and violence appear or when an unfamiliar patient acts out, the most useful strategy is to take a step back. Carers describe that they need time to reflect when the unknown is perceived as frightening. The following quote illustrates how carers can manage their emotions by taking a step back to reflect as a way to regulate and take control over the situation.

*When I don’t have knowledge of how to act or if I don’t know the patient, it may be linked to the fact that it feels scary. I know who he is but not where I have him. You want to get close enough to the patient to get to know them so they don’t remain unknown to you. To take a few steps back can be an excellent strategy on such occasions because it means that I hopefully don’t get in a clinch with him.*

A lack of experience amplifies the likelihood of becoming emotionally overwhelmed, especially with feelings like fear and frustration, since inexperienced carers may not yet have developed a strong sense of confidence in their professional role. Assuming the role of a carer goes beyond mere task management; it also involves seeking a sense of belonging and integration, fostering a feeling of security within the team, and ultimately empowering carers to effectively carry out their responsibilities. Sometimes, fitting in can prove to be a mistake and cause trauma for both the patient and the carer. This may result in carers using coercion and adopting the role of the bulldozer.

*It was a huge and complete failure. The feeling at the beginning was, that we guys are going to fix this, we lived up to the macho norm. But afterwards, I was ashamed of myself and in front of the patient… because it was unnecessary to act in such a violent way in front of other colleagues and patients.*

Carers must be able to become close to the patient, which can be quite stressful. Temporarily fleeing the encounter and then returning is one way of dealing with the situation. Being able to take time away from the patient sometimes and letting a colleague temporarily take over may help. Unfortunately, this is not always possible as it is based on the fact that the patient has to accept that someone else is entering the situation.

*If you yourself are completely exhausted, you cannot bear that the patient continues to just turn to you. You feel your bad mood with all that it means. That’s the problem with this job. Listening to everything is nagging for several days in a row, it consumes one.*

### 3.3. Offering the Patient Emotional Space and Finding Patience

This aspect of fleeing the encounter involves not only dealing with one’s own fear caused by aggressive behavior, protecting oneself, and avoiding harm but also giving the patient time and space to calm down and thereby being able to talk to the carer. This meaning constituent of the phenomenon is characterized by the tension between maintaining or losing patience. Sometimes, the carers are unable to control their emotions and thus risk losing control of themselves and the situation. Sometimes, they go away because they are just losing patience. In such a situation, when they are taking a few steps back, giving the patient space, and making room for themselves, they act like a ballerina. To breathe and calm down can be seen as a strategy that allows the carer to reflect on the situation.

*Sometimes I feel I just can’t handle it; I just need to flee from the fear or the frustration. Of course, it feels wrong I know that it only makes it worse for the patient and my colleagues… but it just becomes too much. At least it gives me time to feel the patient’s situation*.

Encountering a patient who afflicts the carer as a person forces him/her to take a temporary step back to give the patient space to unwind. Coming back to the patient with a fresh perspective entails displaying empathy and the ability to convey trust while encouraging the patient to openly share and navigate their emotions. The cultivation of trust and patience is closely linked with the carer’s presence, where they accept the patient as a fellow human being and acknowledge both the patient’s and their own imperfections. The capacity to manage stressful situations and serve as a role model for the patient is essential in leading by example.

*There is a patient who has an ability to press my buttons, who sort of crawls under my skin. It’s often a threat to me as a person, it’s very unpleasant... Then I have to remind myself that we are all human, he’s probably also scared. I have been in such situations before and feel relatively safe in it. I want him to share his feelings with me even if they are expressed in that way... hopefully he can learn from me.*

Carers emphasize the significance of providing support to others and being the one who possesses the knowledge and capability to manage and respond effectively in various situations. This means that they feel the expectations of remaining calm regardless of whether the situation escalates. Carers stress the importance of maintaining patience and maintaining realistic expectations regarding the patient’s capacity to navigate the situation, cautioning against overestimating the patient’s abilities. In their stories, they also emphasize being able to manage their expectations of themselves and expectations from their colleagues and having reasonable expectations of the patient. Having expectations of oneself means not losing patience and not acting emotionally, being able to control one’s emotions—acting not as a bulldozer but as a ballerina with low affect in the situation.

*We talked about the variations in the staff group. That there were both bulldozers and ballerinas. Then, I remember that we highlighted this with the flexibility, compliance, and how it shows in the communication with the patient.*

## 4. Discussion

Our findings suggest that carers have experienced resistance in the encounters in various ways: they experience fear when confronted with threats, they are forced to make crucial choices alone, they strive to maintain control over their situation, and they accept the tension between remaining in a situation or giving up and escaping.

The results reveal that the essence of the searched aspect of the phenomenon of ‘fleeing the encounter when facing resistance’ means a struggle between acting instinctively primitive and expectantly empathic. This description adds nuances to Martinsen’s person-oriented professionalism in the encounter between carer and patient [34]. In other words, this means understanding one’s position in a specific life context that requires something of oneself as a carer—a concept that means putting the patient in the center, or an orientation toward the other in the encounter. Martinsen [35] also refers to the Danish philosopher Løgstrup’s (1905–1981) concept of ‘untouchable zones’ which should not be violated when encountering the other.

In line with Alvsvåg [34] and Martinsen [35], fleeing the encounter in this context can be understood as a reconnection with oneself through the movement between being there and withdrawing but also by finding a balance in the tension between chaos and stability. A balance that means both being close and keeping one’s distance; to be together and to be alone. Rydenlund et al. [36] state that carer−patient relationships in a forensic context are based on trust, a trust that may arise due to carers having the courage to remain in the situation. According to Bowers [19], carers’ self-regulation of emotional responses, such as fear and anger, corresponds to Angel et al.’s [37] suggestion that being face to face with patients in difficult, in that potentially dangerous situations may trigger the carer’s vulnerability. This situation is closely connected to Løgstrup’s [17] untouchable zone. Løgstrup explained that every human has an untouchable zone, a zone that relates to human integrity and should not be touched or exceeded.

In this study, encounters with patients lead to an ambivalent self-perception. The carer feels insecure and in a situation that risks provoking uncontrollable primitive reactions toward the patient. A situation that culminates when the carer no longer knows where the line goes between care and custody and fear takes over. This aspect of the phenomenon of fleeing from the encounter, shielding oneself from coming to harm or harming the other, involves struggling to find your personal boundaries and having the courage to face the patient. In their stories, carers describe that they become aware of how powerful and contagious the patient’s actions actually are. It affects the entire staff group and is something against which you cannot defend. When a threatening situation arises, the fight or flight instinct reacts—a primitive response that must be regulated so as not to allow the impulse to take over the situation.

In such situations, carers are usually alone with the patient and have no one to turn to. The carer has to withdraw from the patient in order to have the opportunity to reflect. They see fleeing the situation as a strategy to control their emotions. The situation arouses fear in the carer, a fear that affects their actions. Other studies point out that threatening situations and fear can affect the carer’s identity by leading to a negative self-perception that affects the carer’s actions [38,39].

The meaning constituent finding one’s emotional balance or being exposed, means to be distanced, like a bulldozer, or compassionate and caring like a ballerina. This aspect is characterized by a tension between knowledge and ignorance, that is, being between conflicting feelings that are linked to insecurity in one’s professional role, logically, a view about local, bodily, and experience-based knowledge. A key strategy in forensic psychiatry to establish a caring relationship is transparency and openness from the carer [40]. According to Martinsen [41], the phenomenological starting point is that our openness to the world shall be taken seriously (i.e., that we are open-minded, attentive, and concerned in the place of impressions).

To grasp and understand what we are touched by and make our impression of the expression conscious, we must acquire distance, a distance that provides for understanding [35]. However, this does not mean going out of the situation to see it from the outside. In the impression, we are open to ourselves and what the impression gives [27].

This aspect of the phenomenon of fleeing from the situation involves the carers’ doubt about their professional identity. Identity is, according to Watson [42], a narrative construction about who a person is in relation to the life one has lived and the person’s wishes for the future. The identity is individual but also cultural because humans are part of social and historical contexts. When something fatal happens that conflicts with your expectations, the self’s vulnerability emerges, and meaninglessness can arise.

The findings also point to the feeling of shame. Not living up to one’s own expected professional identity can be considered degrading and a sign of weakness that one is no longer able to take care of difficult situations that have arisen at work. In the carers’ stories, the shame seems to go beyond the interaction with the patient and be linked to the relationship with oneself and that fleeing the encounter collides with who one thinks one should be.

According to Martinsen [35], shame can be seen as something positive, as it forces one into a critical way of thinking and into self-reflection on one’s action.

The meaning constituent offering the patient emotional space and finding patience means not only dealing with one’s own fear, protecting oneself, and avoiding harm, but also giving the patient time and space to calm down and therefore be able to talk to the carer. The phenomenon of fleeing the situation is characterized by the tension between maintaining or losing patience. Sometimes, carers cannot control their emotions and thus risk losing control of themselves and the situation. It is almost as if the carer’s patience is running out and they are forced to back away to avoid acting based on their emotions. In such a situation, when they take a few steps back, the carer gives both parties space to breathe and calm down. However, being at a distance means risking presenting oneself as uncaring and insensitive in the eyes of the patient [37].

Patience is generally considered to be a moral quality, an ability to wait or endure difficulties while maintaining calm in the face of external stress without giving in to uncontrolled emotional outbursts. Unlike anxiety, melancholy, happiness, etc., which, according to Heidegger [31], constitute basic moods, patience is directed toward a specific object. It is reasonable to assume that patience relates to courage, the courage to endure and overcome the fear of being a disappointment and/or a colleague that others cannot trust (i.e., a carer who cannot live up to the demands of those around them).

Encountering a patient who affects the carer has a negative effect on the carer who is forced to take a temporary step back to give the patient space to unwind. Promoting patience requires stability, perseverance, and specific skills related to staying calm [43]. Returning to the patient with renewed vigor means being understanding. In the situation, expressing trust and inviting the patient to share and manage their feelings develops trust between the parties [44]. Being able to handle a stressful situation and lead by example means appearing as a role model for the patient.

### 4.1. Implications for Practice

A lack of understanding of carers’ experiences in encounters may lead to exclusion and challenge the carer’s professional identity and integrity. By focusing on the vulnerability that the encounter may entail and exploring the carers’ subjective experiences of the encounter with the patient, their vulnerability and courage can reasonably be turned into a strength in the encounter, and they can approach the patient with an open and responsive attitude. In a forensic psychiatric care context, the carer is a key person when establishing a carer–patient relationship. This study contributes knowledge that encourages both patient-focused care and increased efforts for staff self-care. Without insight into or understanding of one’s emotional life, the ability to provide good care to others is also challenged. Difficult patient encounters are an integral part of everyday life in forensic care, and carers need both the acceptance of and reflection on their actions. If it can be arranged for carers to have an arena where they can safely and actively reflect on experiences from patient encounters, where there is a potential for promoting personal development. This not only enriches carers, who can gain an increased understanding of themselves, but also the quality of care, because the carer, to a greater extent, gains insight into the patient’s expression of suffering. We suggest that the management of the department has a responsibility to facilitate such reflections, both to ensure patients’ proper care as well as to protect and strengthen their employees.

### 4.2. Methodological Considerations

Data collection was planned before the detailed literature search and a description of the analytical steps was established. Through an open interview with broad follow-up questions, the participants told their experiences from their natural attitudes, which gave a lot of variations in the subject. The rich descriptions made it possible to present the meaning structures of the phenomenon by being faithful and to present the carers’ experiences in the analysis.

The first author’s prior knowledge of and pre-existing comprehension regarding the phenomenon and context could be perceived as both advantageous and cumbersome. This familiarity proved advantageous in that it enabled participants to express themselves openly and truthfully. Conversely, it posed a challenge when attempting to critically examine one’s inherent perspective. Consequently, this challenge was alleviated by the co-authors due to their lack of familiarity with the context being studied. We found that multiple discussions facilitated the analysis process. Ultimately, this shared contemplation led to a collective comprehension. By not trying to understand too quickly, we were able to continue to wonder about the implications of the data and convey impressions that prompted discussions at a scientific level in the research group. This strengthened the objectivity, validity, and generalization of the results [27,28]. We wish to highlight the absence of recent studies regarding how carers in this context comprehend and manage their own emotions during interactions with patients [25], which poses a challenge when attempting to compare our findings with previous research.

## 5. Conclusions

The essential meaning of fleeing the encounter when facing resistance turned out to be a complex phenomenon intertwined with the carers’ self-perception as professional carers. The carers’ experience was that an ordinary patient encounter could suddenly turn into something threatening and dangerous, causing a dilemma. They knew that the encounter could contribute positively to the long-term nature of the relationship but, at the same time, it was perceived as an unbearable situation. Since carers are in a context where they cannot avoid patient encounters, negative encounters affect their self-perception and evoke shame and feelings of self-blame. The effect of a changed encounter was thus not only a matter of endurance but was also associated with the carers’ self-perception as professionals, their patience, and a concern about not having control over their profession.

In a forensic psychiatric care context, the carer is a key person whose task is to shape the care relationship, which requires an attitude on the part of the carer that recognizes not only the patient’s lifeworld but also their own.

## Figures and Tables

**Table 1:** Example of the analysis process.

Reading and Rereading of Transcripts		Grouping of Meaning Units into Clusters	Assembling the Clusters of Meaning in Patterns
Interview Text	Content	Meaning Unit	Meaning Constituent
*When we were standing in that elevator and the patient raised his voice and became aggressive, I could feel the tension rising. For me, the fact that he was such a physically capable man frightened me, I did not know how to handle it.*	In an elevator with a patient, a sudden aggressive behavior heightened tension. His physical strength left the carer feeling fearful and unsure about how to manage the situation	Self-preservation and avoiding harm to others	Shielding Oneself From Coming to Harm or Harming the Other
*It was a huge and complete failure. The feeling at the beginning was, that we guys are going to fix this, we lived up to the macho norm. But afterwards, I was ashamed of myself and in front of the patient because it was unnecessary to act in such a violent way in front of other colleagues and patients.*	The carer was ashamed of having used superior force and violence because it was unnecessary in the situation and worked against its purpose	Reacting instinctively like a bulldozer	Finding One’s Emotional Balance or Being Exposed
*He just kept nagging and nagging, and at that moment I felt that the frustration took over. Then, I was thankful for my colleague that he stepped in and took over the conversation. Then the patient directly became calmer*.	Persistent nagging led to frustration, but relief came when a colleague intervened and took control of the conversation which calmed the patient	Giving the patient room to express emotions and being patient with them	Offering the Patient Emotional Space and Finding Patience

## Data Availability

Available on request to corresponding author.
